# High Molecular Prevalence of *Anaplasma* spp. in Cattle from the Sertão and Agreste Regions, Northeast Brazil

**DOI:** 10.1007/s11686-026-01322-0

**Published:** 2026-06-06

**Authors:** Leila Maria Rosa dos Santos, Matheus Dias Cordeiro, Maiara Vasconcelos Monteiro, Marina de Paula de Almeida, Juliano Martins Santiago, Claudia Bezerra da Silva, Bruna de Azevedo Baêta, Jonatas Campos de Almeida

**Affiliations:** 1 Laboratory of Parasitology and Parasitic Diseases of Domestic Animals, Center for Engineering and Agricultural Sciences, Federal University of Alagoas, Viçosa, Alagoas Brazil; 2 Laboratory of Host-Parasite Interaction Studies, Institute of Veterinary Medicine, Federal Rural University of Rio de Janeiro, Seropédica, Rio de Janeiro, Brazil; 3 Maurício de Nassau University Center, Recife Campus, Recife, Pernambuco Brazil; 4 Academic Unit of Serra Talhada, Federal Rural University of Pernambuco, Serra Talhada, Pernambuco Brazil; 5 Preventive Veterinary Medicine Sector, Department of Veterinary Medicine, School of Veterinary Medicine and Animal Science, Federal University of Goiás, Goiânia, Brazil

**Keywords:** Enzootic stability, PCR, Caatinga, Anaplasmosis, *msp4*

## Abstract

Bovine anaplasmosis is a tick-borne rickettsial disease of significant sanitary and economic importance to livestock, particularly in tropical and subtropical regions, and is associated with high morbidity and reduced productivity. However, data on its molecular prevalence in cattle from semiarid and transitional areas of the Northeast region of Brazil remain scarce. This study aimed to determine the prevalence of *Anaplasma* spp. in cattle from smallholder farms located in the Sertão and Agreste mesoregions of the states of Paraíba and Pernambuco, Brazil. A total of 380 whole blood samples from cattle were analyzed by conventional PCR targeting the *msp4* gene, following confirmation of DNA extraction by amplification of the endogenous *GAPDH* gene. *Anaplasma* spp. was detected in 92.1% of the cattle, with no significant association with sex, age group, breed, production system, or level of tick infestation. Positive cattle exhibited significantly lower hematocrit values compared to negative animals (*p* = 0.0087). These results represent the first report of molecular detection of *Anaplasma* spp. in cattle from the states of Pernambuco and Paraíba and suggest the presence of enzootic stability microregions in the Sertão and Agreste mesoregions of Northeast Brazil.

## Introduction

Infections caused by rickettsiae of the genus *Anaplasma* constitute a major sanitary challenge for global livestock production. Transmitted by ticks, these agents compromise herd health and productivity. Anaplasmosis has been recognized for over a century as a significant cause of economic losses in various regions, particularly when associated with babesiosis [1–4]. The high morbidity characteristic of anaplasmosis results in decreased productivity and can be lethal in susceptible cattle herds located in areas of enzootic instability [5]. Among the described species, *A. marginale* is of greatest veterinary importance; however, other species, such as *A. centrale* and *A. bovis*, have also been associated with disease [6].

Widely distributed in tropical and subtropical countries, bovine anaplasmosis is considered endemic in Latin American and Caribbean countries, where environmental conditions favor the life cycle of arthropod vectors [7]. In Brazil, biological transmission occurs primarily through the ixodid tick *Rhipicephalus (Boophilus) microplus*; however, mechanical transmission can occur via hematophagous flies of the genus *Tabanus* and *Stomoxys calcitrans*, as well as through contaminated fomites [7, 8]. Cattle that recover from anaplasmosis become chronic carriers, maintaining persistent subclinical infection. These animals serve as reservoirs and contribute to maintaining the bacterium within the herd [9].

The epidemiological situation of bovine anaplasmosis in Brazilian territory is heterogeneous, marked by the coexistence of areas of enzootic stability and instability, resulting from the climatic diversity present in the country’s different biomes [10]. The Northeast region comprises four biomes, with the Caatinga being the most prominent. This biome is characterized by a semiarid climate and the presence of distinct dry and rainy seasons throughout the year. These characteristics may limit the biological cycle of vectors, favoring the occurrence of clinical outbreaks in animals not previously exposed to rickettsiae [11]. In transitional areas such as the Agreste, disease epidemiology is variable due to the presence of an area of enzootic stability, such as the Atlantic Forest, and an area considered unstable, such as the Caatinga [10].

Previous epidemiological studies conducted in Brazil have demonstrated substantial variation in the occurrence of bovine anaplasmosis according to climatic conditions, vector dynamics, and production systems, with distinct epidemiological patterns observed among enzootic stability and instability areas [12–14]. Despite the recognized importance of bovine anaplasmosis in tropical and semiarid environments, molecular epidemiological investigations remain limited in several regions of Northeast Brazil, particularly in areas characterized by climatic transition and heterogeneous vector pressure. Therefore, investigating the occurrence of *Anaplasma* spp. in cattle from semiarid and transitional environments may contribute to a better understanding of the epidemiological dynamics of bovine anaplasmosis in Brazil.

Identification of hemoparasites in blood smears is the oldest and most widely used diagnostic method in clinical routine for diagnosing tick-borne infectious diseases [15]. However, its sensitivity is limited, particularly in chronic carriers or animals with low rickettsemia, a common scenario in endemic areas. Thus, molecular techniques for amplification of genetic material have assumed a fundamental role in infection diagnosis due to their higher specificity and sensitivity [16].

## Materials and Methods

### Study Area

**Fig. 1:**
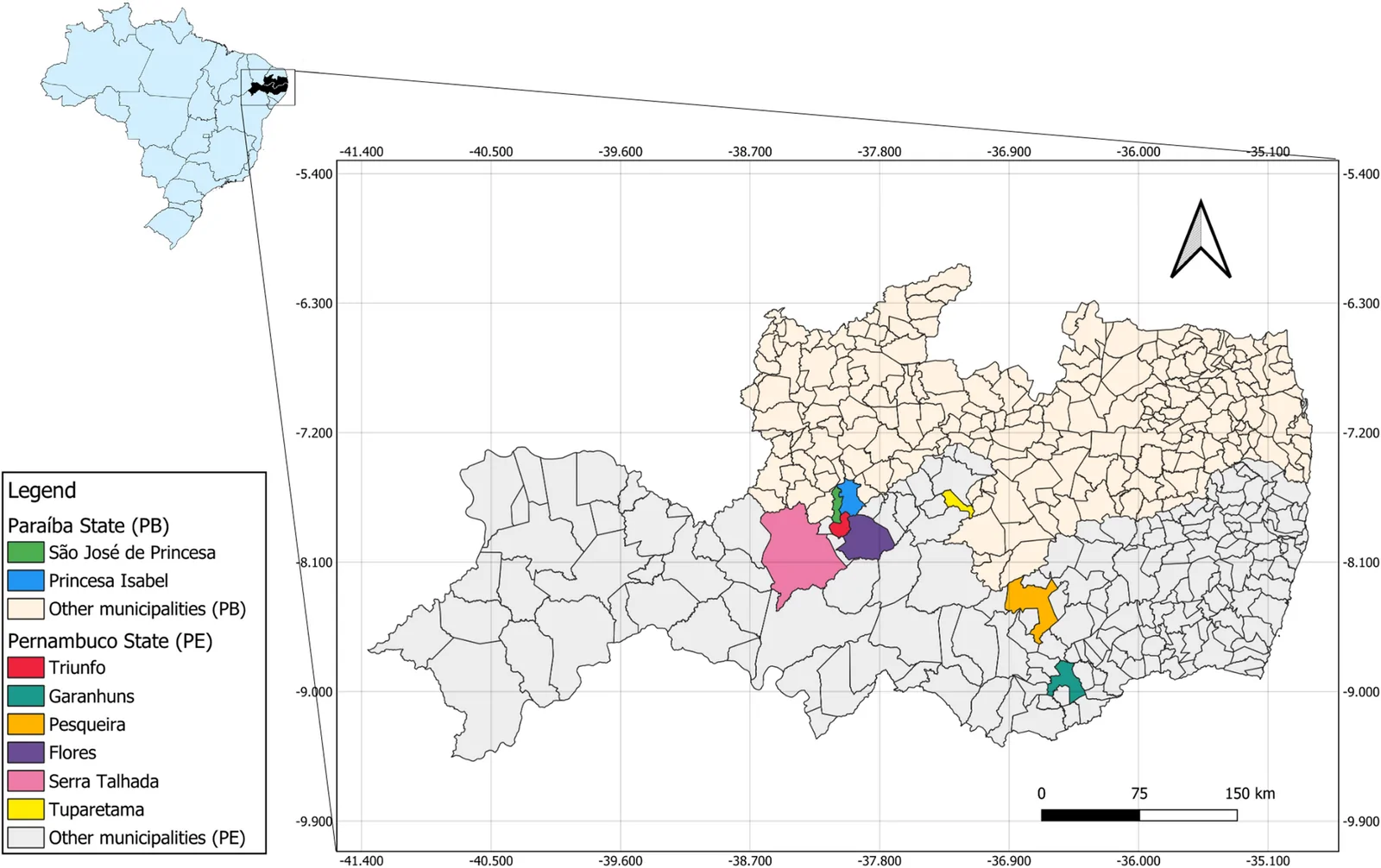
Map of sample collection municipalities for the molecular prevalence study of *Anaplasma* spp. in the Sertão and Agreste regions of Northeast Brazil, generated using QGIS 3.40.11 software

This study was conducted on smallholder farms in municipalities of the Sertão and Agreste regions of Northeast Brazil, classified by production system (extensive or semi-extensive). In Paraíba, five farms were selected from the Serra do Teixeira microregion, within the Sertão Paraibano mesoregion, with three in São José de Princesa (7°47’10.9” S; 38°06’24.2” W) and two in Princesa Isabel (7°44’12.4” S; 37°59’16.4” W). In Pernambuco, 11 farms from the Pajeú microregion in the Sertão Pernambucano mesoregion were included, located in the following municipalities: one in Flores (7°51’26.6” S; 37°58’19.6” W), one in Serra Talhada (7°51’05.1” S; 38°33’11.6” W), six in Triunfo (7°50’09.2” S; 38°06’14.3” W), and five in Tuparetama (7°36’50.6” S; 37°18’43.9” W); as well as farms from the Agreste Pernambucano mesoregion, with one farm located in the municipality of Pesqueira (8°33’05.3” S; 36°38’53.3” W) in the Vale do Ipojuca microregion, and two in Garanhuns (8°53’26.6” S; 36°29’33.8” W), in the Garanhuns microregion. These municipalities represent a cross-section of the northeastern semiarid region, encompassing areas of the Sertão and Agreste (Fig. 1).

The predominant biome in the study area is the Caatinga, characterized by xerophilous vegetation and a semiarid climate, with annual average temperatures between 23 °C and 27 °C and precipitation ranging from 500 to 900 mm, with rainfall concentrated between February and May [17, 18]. In areas of higher altitude, such as Triunfo and Garanhuns, there are transitional stretches to the Atlantic Forest and the occurrence of so-called “altitude wetlands” (brejos de altitude), characterized by annual average temperatures of 20 °C to 24 °C and average annual precipitation of 800 to 1000 mm, influenced by relief and the Borborema Plateau [17, 18]. Vegetation exhibits phytogeographic transitional formations, with plant foliage sprouting during rainy periods and falling during dry periods [19]. The municipalities in Paraíba are located in areas of high altitude, in transitional zones with the Borborema Plateau, conferring milder average temperatures and higher precipitation due to their relief and altitude [18, 20].

### Sampling and Samples

Minimum sample size was calculated using the formula for simple random sampling [21], considering a 95% confidence level (Z = 1.96), an expected prevalence of 50% to provide the largest possible sample value, and a 5% margin of error, resulting in an estimated minimum sample size of 384 individuals. Animals were subjected to physical examination before blood collection, with recording of information such as breed, sex, age, level of tick infestation, and ocular mucosa coloration. Animals were divided into two age groups (≤ 24 months and > 24 months), with age estimated through dental chronology of incisors [22]. The level of tick infestation was estimated using the method described by Wharton et al. (1970), in which ticks are counted on one side of the animal, and the total burden is obtained by doubling this value [23]. Based on the estimated total tick burden, animals were categorized using a qualitative scale as follows: absent (no ticks), low (1–50 ticks), medium (51–100 ticks), and high (> 100 ticks). Samples of 4 mL of whole blood were collected from cattle by jugular venipuncture into tubes containing ethylenediaminetetraacetic acid (EDTA). The collected volume was used for the determination of packed cell volume using the microhematocrit technique, with the remainder aliquoted into polypropylene tubes and stored at − 20 °C until molecular analysis.

### Molecular Analysis

Extraction of genetic material from blood samples was performed following the phenol–chloroform protocol described by Sambrook et al. [24]. Total DNA samples obtained were stored at − 20 °C until subsequent steps. To confirm extraction efficiency and prevent possible false-negative results, conventional polymerase chain reaction (PCR) was performed to amplify a fragment of the mammalian glyceraldehyde-3-phosphate dehydrogenase (*GAPDH*) gene, using the primer pairs GAPDH-F (5’-CCTTCATTGACCTCAACTACAT-3’) and GAPDH-R (5’-CCAAAGTTGTCATGGATGACC-3’), according to the protocol of Birkenheuer et al. [25]. Detection of *Anaplasma* spp. was performed by conventional PCR to amplify an 831 bp fragment of the gene encoding major surface protein 4 (msp4), using primers msp4-F (5’- TTGTTTACAGGGGGCCTGTC-3’) and msp4-R (5’- GAACAGGAATCTTGCTCCAAG-3’), according to the protocol of Mahmood et al. [26].

### Statistical and Epidemiological Analysis

Initially, descriptive analysis of the variables was performed, calculating absolute and relative frequencies for categorical variables (state, production system, breed, sex, age, and degree of infestation) and measures of central tendency and dispersion (mean, standard deviation, median, and interquartile range) for continuous variables (hematocrit value). Normality of continuous variables was assessed using the Shapiro–Wilk test, while homogeneity of variances between groups was verified using Levene’s test [27]. Molecular prevalence of *Anaplasma* spp. was calculated based on samples positive for the *msp4* gene relative to the total samples analyzed, accompanied by its respective 95% confidence interval (95% CI) based on the Wilson method. The association between PCR positivity and explanatory variables was evaluated through bivariate analysis. For categorical variables, Pearson’s chi-square test or Fisher’s exact test was used. For continuous variables, Student’s t-test (normal distributions and homogeneous variances) or the Mann–Whitney test (non-parametric distributions or heterogeneous variances) was employed [28]. Variables with *p*-value ≤ 0.20 in the bivariate analysis were selected for inclusion in the multivariate binary logistic regression model, aiming to identify factors independently associated with *Anaplasma* spp. infection. The strength of association was expressed as an odds ratio (OR) with 95% confidence intervals. The presence of multicollinearity among independent variables was verified using the variance inflation factor (VIF), with VIF > 10 considered the exclusion threshold [29, 30]. All statistical and epidemiological analyses were performed using RStudio software. The significance level adopted was 5%.

## Results

Whole blood was collected from 384 cattle on 21 farms in the Sertão and Agreste regions. Of the 384 samples collected, 380 resulted in amplification of the mammalian endogenous *GAPDH* gene and were therefore included in the study. Molecular prevalence of *Anaplasma* spp. was 92.1% (350/380; 95% CI 88.95–94.41%), estimated using the Wilson method, considered robust for high proportions. The distribution of positive and negative animals for *Anaplasma* spp. in relation to the epidemiological variables evaluated is presented in Table 1.

**Table 1:** Univariate analysis of the association between categorical variables and PCR positivity for *Anaplasma* spp. in cattle from Pernambuco and Paraíba states, Northeast Brazil

Variable	*N*	Positive (%)	Univariate (*p*-value)
	380	350 (92.1%)	
*Sex*			
Male	103	96 (93.2%)	0.628
Female	277	254 (91.7%)	
*Age*			
> 24 months	210	195 (92.8%)	0.546
≤ 24 months	170	155 (91.2%)	
*Breed composition*			
Taurine	41	39 (95.1%)	0.623
Zebu	56	53 (94.6%)	
Crossbred	283	258 (91.2%)	
*Production system*			
Semi-extensive	149	139 (93.3%)	0.492
Extensive	231	211 (91.3%)	
*State*			
Pernambuco	306	283 (92.5%)	0.578
Paraíba	74	67 (90.5%)	
*Level of infestation*			
High	10	10 (100.0%)	0.654
Medium	24	21 (87.5%)	
Low	249	229 (92.0%)	
Absent	97	90 (92.8%)	

**Fig. 2:**
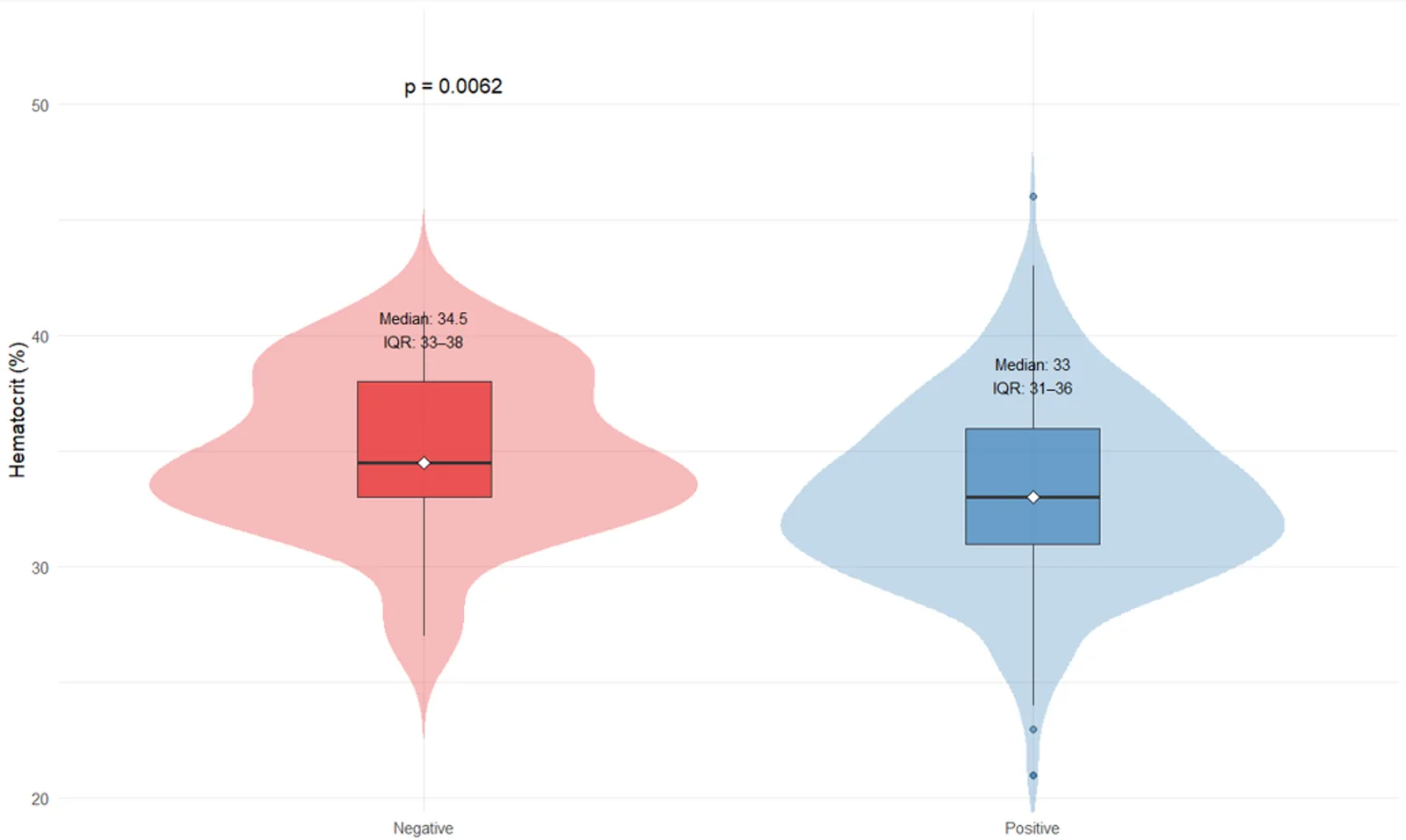
Distribution of hematocrit in negative and positive animals for *Anaplasma* spp. by polymerase chain reaction amplifying the *msp4* gene in cattle from Pernambuco and Paraíba states, Northeast Brazil

None of the variables presented a statistically significant association with PCR positivity (*p* > 0.20). Consequently, no variables were selected for the multivariate binary logistic regression model. These results indicate that, in the context of this sample, no epidemiological or demographic factor evaluated presented an independent influence on infection. Two positive animals exhibited pale mucous membranes, while the remainder exhibited normal coloration. Regarding hematocrit, positive animals exhibited a non-normal distribution and heterogeneous variances compared to negative animals, justifying the use of the non-parametric Mann–Whitney test. A significant difference was observed between the groups (*p* = 0.0087), with a median of 33.1% (IQR 31.0–36.0) for positive animals and 34.9% (IQR 32.5–36.8) for negative animals for *Anaplasma* spp. (Fig. 2). These findings suggest that *Anaplasma* spp. infection is associated with reduced hematocrit, reflecting biological effects in infected animals.

## Discussion

The high molecular prevalence of *Anaplasma* spp. found (92.1%; 350/380; 95% CI 88.95–94.41%) suggests an enzootic stability scenario. Although epidemiological studies addressing the prevalence of anti-*Anaplasma* spp. antibodies in cattle from the Northeast exist, or even detecting the agent’s presence through blood smears or directly in ticks, there remains a research gap in estimating the occurrence or prevalence of the agent in regional herds, particularly in Pernambuco and Paraíba states, Brazil. Thus, to date, this is the first study reporting direct detection of *Anaplasma* spp. by PCR in cattle from Pernambuco and Paraíba states, Brazil.

Paraíba state has a history of bovine anaplasmosis outbreaks with significant morbidity and mortality, particularly in the Sertão, with cases concentrated between the end of the rainy season and the beginning of the dry season, a period that favors proliferation of the vector *R. microplus* [13, 31]. In the first decade of the 21st century, 38 cases of bovine babesiosis and anaplasmosis complex were diagnosed by the Veterinary Hospital of the Federal University of Campina Grande in Paraíba; of these, 23 were caused exclusively by *A. marginale* and nine by coinfection with *Babesia* sp [13]. Most outbreaks occurred in regions of higher altitude and proximity to perennial rivers, suggesting these areas as enzootic instability zones. The role of mechanical transmission in these regions is also notable [13].

The Paraíba municipalities analyzed in this study (São José de Princesa and Princesa Isabel) possess geographic and environmental characteristics similar to those analyzed in studies by Costa and collaborators (2011) in the Paraíba Sertão [13]. All municipalities are located in the semiarid region, with predominance of the Caatinga biome, semiarid climate, low rainfall, and presence of high altitudes. These environmental conditions favor the formation of microclimates suitable for biological vector development, especially during the rainy period when water bodies remain active [10].

In Pernambuco state, the relevance of bovine anaplasmosis as a sanitary problem was demonstrated in a retrospective hospital-based study conducted at the Bovine Clinic of Garanhuns, Federal Rural University of Pernambuco [32]. In that study, the disease was the main tick-borne infectious condition diagnosed in the last decade and was commonly associated with anemia, jaundice, and productive losses. The authors reported that prolonged drought between 2012 and 2016 reduced case occurrence, whereas subsequent periods of increased rainfall and milder temperatures favored tick cycles and bovine anaplasmosis occurrence. In the present study, despite the high prevalence, only two positive animals exhibited pale mucous membranes, while the remainder showed normal coloration, indicating the absence of classic clinical signs in most infected cattle.

Despite the frequently subclinical presentation observed in endemic areas, bovine anaplasmosis remains an important sanitary challenge for livestock production due to its association with anemia, weight loss, reduced productivity, mortality in susceptible animals, and economic losses related to treatment and vector control [8, 12]. Additionally, chronically infected cattle may persist as reservoirs for long periods, contributing to the maintenance and dissemination of the agent within herds [8].

A study conducted by Moura et al. (2024) on cattle from Catimbau National Park, a semiarid region in central Pernambuco representative of the Caatinga biome, demonstrated 34% seropositivity (16/47) for anti-*A. marginale* antibodies, with no association with risk factors [14]. Similarly, research conducted in Petrolina and Ouricuri, municipalities with predominance of the Caatinga biome, indicated seroprevalences of 35% and 45.5%, respectively [33]. These studies, restricted to serological techniques such as IFAT and ELISA, have historically characterized these semiarid regions as enzootic instability areas.

However, in the present study, which encompasses municipalities within both the Caatinga and Agreste regions, molecular analysis demonstrated a prevalence of 92.1% of the agent in the analyzed cattle. The prevalence detected by PCR suggests a possible transition to enzootic stability in microregions previously considered unstable, or even the greater sensitivity and specificity of molecular techniques compared to serological techniques, allowing gene amplification even in patients with low rickettsemia [34].

Although semiarid regions are traditionally recognized as enzootic instability areas, since climatic factors limit the *R. microplus* population and, consequently, rickettsia circulation [10], the results of the present study demonstrate a high prevalence of *Anaplasma* spp. in municipalities of the Agreste and Sertão. Notably, among the examined cattle, absence or low tick infestation predominated (Table 1), reinforcing the hypothesis that mechanical transmission by hematophagous insects [35], or the presence of chronic carriers and inadequate management practices, such as the shared use of non-sterilized instruments, also contribute to infection persistence in herds, regardless of infestation by the rickettsia’s main vector [8].

Although cattle of all ages are susceptible to anaplasmosis, studies demonstrate a higher prevalence in animals older than 24 months [13, 32]. However, in this study, no significant difference was observed between groups. Regarding subspecies, several authors reported that cattle with greater taurine composition are more susceptible to *R. microplus* infestation and, consequently, exhibit a higher frequency of *A. marginale* infection, while zebu and crossbred animals tend to be more resistant to tick-borne infectious diseases [36]. In this study, however, no association was observed between subspecies and crossbreeds and positivity for *Anaplasma* spp. (*p* = 0.623), suggesting intense and homogeneous exposure to the agent in the evaluated herds.

In this study, both positive and negative animals for *Anaplasma* spp. exhibited hematocrit values within the reference range [37]; however, positive animals presented a lower median than negative cattle (*p* = 0.0087). This difference is consistent with the extravascular removal of infected erythrocytes described in *Anaplasma* spp. infection. Bovine anaplasmosis is characterized by extravascular hemolytic anemia, with marked hematocrit reduction in clinical cases [38]. In enzootic stability areas, however, cattle may remain chronic carriers with low rickettsemia and mild hematological alterations, characterizing subclinical anaplasmosis. These findings are compatible with the high prevalence observed and suggest enzootic stability sub-areas in the Sertão of Paraíba and Pernambuco, as well as in the Agreste of Pernambuco state.

## Conclusion

This study represents the first report of molecular detection of *Anaplasma* spp. in cattle from the Sertão and Agreste regions of Paraíba and Pernambuco states, Northeast Brazil, indicating widespread rickettsia circulation in regions previously categorized as enzootic instability areas and suggesting the existence of enzootic stability microregions in semiarid and Agreste environments in Northeast Brazil.

## Data Availability

No datasets were generated or analysed during the current study.
